# Bringing an Effective Behavioral Weight Loss Intervention for People With Serious Mental Illness to Scale

**DOI:** 10.3389/fpsyt.2018.00604

**Published:** 2018-11-20

**Authors:** Emma E. McGinty, Kimberly A. Gudzune, Arlene Dalcin, Gerald J Jerome, Faith Dickerson, Joseph Gennusa, Stacy Goldsholl, Deborah Young, Gail L. Daumit

**Affiliations:** ^1^Department of Health Policy and Management, Johns Hopkins Bloomberg School of Public Health, Baltimore, MD, United States; ^2^Division of General Internal Medicine, Johns Hopkins University School of Medicine, Baltimore, MD, United States; ^3^Department of Kinesiology, Towson University, Towson, MD, United States; ^4^Sheppard Pratt Health System, Baltimore, MD, United States; ^5^Department of Research & Evaluation, Kaiser Permanente Southern California, Pasadena, CA, United States

**Keywords:** exercise, diet, obesity, weight loss, serious mental health conditions

## Abstract

People with serious mental illnesses (SMIs) die 10–20 years earlier than the general population, mainly due to cardiovascular disease. Obesity is a key driver of cardiovascular risk in this group. Because behavioral weight loss interventions tailored to the needs of people with SMI have been shown to lead to clinically significant weight loss, achieving widespread implementation of these interventions is a public health priority. In this Perspective, we consider strategies for scaling the ACHIEVE behavioral weight loss intervention for people with SMI, shown to be effective in a randomized clinical trial (RCT), to mental health programs in the U.S. and internationally. Given the barriers to high-fidelity implementation of the complex, multi-component ACHIEVE intervention in often under-resourced mental health programs, we posit that substantial additional work is needed to realize the full public health potential of this intervention for people with SMI. We discuss considerations for successful “scale-up,” or efforts to expand ACHIEVE to similar settings and populations as those included in the RCT, and “scale-out,” or efforts to expand the intervention to different mental health program settings/sub-populations with SMI. For both, we focus on considerations related (1) intervention adaptation and (2) implementation strategy development, highlighting four key domains of implementation strategies that we believe need to be developed and tested: staff capacity building, leadership engagement, organizational change, and policy strategies. We conclude with discussion of the types of future research needed to support ACHIEVE scale-up/out, including hybrid trial designs testing the effectiveness of intervention adaptations and/or implementations strategies.

## Introduction

People with serious mental illnesses (SMIs) die 10–20 years earlier than the general population, primarily due to cardiovascular disease, and (1–6) obesity is one of the driving forces of cardiovascular risk in this group (7). As in the overall population, poor diet and low rates of physical activity are key contributors to obesity among people with SMI (8–16). In addition, most people with SMI require long-term use of psychotropic medications, which can cause weight gain (17–21). A 2015 review concluded that tailored behavioral weight loss interventions can lead to clinically significant weight loss in this group (22). Spreading implementation of these evidence-based interventions to the mental health organizations that serve people with SMI is a priority for the field.

One intervention shown to be effective in a randomized clinical trial (RCT) is the ACHIEVE behavioral weight loss intervention for people with SMI (23). In this Perspective, we consider strategies for widespread implementation of ACHIEVE. We posit that substantial additional work is needed to realize the full public health potential of this behavioral weight loss intervention for people with SMI.

## Achieve RCT study setting and population

The ACHIEVE RCT has been described in detail previously and is summarized here (23, 24). The ACHIEVE RCT was conducted with adults with SMI participating in one of 10 psychiatric rehabilitation programs in Maryland. To be eligible for psychiatric rehabilitation services in Maryland, individuals must have impaired role functioning resulting in a need for rehabilitation services to develop or restore independent living skills. Overweight and obese adults with SMI ages 18+ were eligible to participate in the ACHIEVE RCT.

The study setting and population were intentionally selected and eligibility criteria were minimized in order to represent realistic mental health programs and diverse participants with SMI (25). The sample included people of diverse race/ethnicities (56% white, 38% black, 6% other race, 5% Hispanic ethnicity) and 52% of participants had a history of substance use, a common co-morbidity among people with SMI (23).

## Achieve behavioral weight-loss intervention

The RCT evaluating the behavioral weight loss intervention found that ACHIEVE resulted in mean weight loss of seven lbs. over 18 months; this was the first trial to demonstrate that a behavioral intervention leads to clinically significant weight loss at 18 months among persons with SMI (23). In addition to ACHIEVE, one other behavioral weight loss intervention has been shown in a RCT to lead to weight loss among people with SMI: the STRIDE intervention resulted in mean weight loss of 5.7 lbs. over 12 months.^27^ A RCT testing the InSHAPE behavioral weight loss intervention for people with SMI showed no effects of the intervention on weight loss among people with SMI over 12 months, but found 24% of participants with SMI randomized to the intervention had both an increase of at least 50 meters on the 6 min walk physical activity measure and a 5% or greater reduction in body weight, compared to 9% of control group participants (26). The ACHIEVE (23), STRIDE (27), and InSHAPE (26) RCT results have all been published previously. Scale-up of multiple effective behavioral weight loss interventions for people with SMI would be desirable. We focus on the ACHIEVE intervention here as a case study. ACHIEVE was developed based on a comprehensive lifestyle intervention for the general population shown to be effective in the PREMIER Trial (28). ACHIEVE integrates social cognitive, behavioral self-management and relapse prevention model theories, and fits well in the psychiatric rehabilitation framework of skill building and environmental supports.

ACHIEVE intervention components are shown in Table 1. Participants with SMI were encouraged to set a goal of 10 lbs. weight loss in 18 months; this goal was tailored as needed to individuals' preferences. The intervention was designed to address the neurocognitive deficits in working memory, verbal memory and executive function that are common in persons with SMI including teaching material in small, highly structured, repeated content units, emphasizing behavioral rehearsal and role-playing, hands-on activities, tailored simple self-monitoring, environmental prompts, and reinforcements. A 5–8th grade reading level was used for program materials. The ACHIEVE curriculum focused on repeated delivery of six key behavioral recommendations (e.g., “avoid sugary drinks”; see Table 1).

**Table 1:** ACHIEVE behavioral weight loss intervention core components.

**Intervention components**	**Type and frequency of contact**
Goal setting	Weight loss goal 10 lbs. in 18 months (tailored to individual)
Key behavioral recommendations	Avoid sugary drinks; avoid junk food; eat five servings of fruits/vegetables per day; portion control; smart snack habits/ and regular physical activity.
**INTERVENTION CONTACTS**	
Group weight management session	Months 1–6 45 min session 1x per week, 3 of 4 weeks, led by study interventionist Months 7–18 45 min session 1x per month, led by study interventionist
Individual weight management session	Months 1–18 15–20 min session, 1x per month, led by study interventionist
Group exercise classes	Months 1–6 50 min session, 3x per week, led by study interventionist. Months 7–12 50 min session, 3x per week led by study interventionist (2x per week) or psychiatric rehabilitation program staff, with video-assistance (1x per week). Months 13–18 50 min session, 3x per week led by study interventionist (1x per week) or psychiatric rehabilitation program staff, with video-assistance (2x per week).
Brief Weigh-ins	Months 1–6 1x per week Months 7–18 2x per month
Self-monitoring “tracker”	Participants fill out a simple tracking tool that records the number of servings of fruits and vegetables, whether they exercised outside of the group classes above for 30 min, and whether they drank sugar drinks; ate junk food; achieved smart portions; and achieved smart snacking goals.
Incentive program	Study participants were rewarded for participation in group and individual weight management sessions and group exercise classes with a point system, which participants could trade points for small prizes (e.g., athletic socks or headphones).
Environmental prompts	Reminders to be used at home: high impact behavioral goals on laminated card, refrigerator magnets, pre-printed grocery lists, water bottle, measuring cups, lunch bags.

The study interventionist delivered the weight-management content through a 45 min group weight management session 3 weeks/month for the first 6 months and then monthly for months seven-18. While a portion of each session was didactic, the groups were interactive and emphasized goal-setting, role-playing and behavioral rehearsal of key concepts. Once-monthly individual weight management sessions with the study interventionist provided the opportunity for individualized cognitive tailoring and a personalized approach, where participants received feedback on weight loss progress, set and adjusted goals, and problem-solved as needed. For the duration of the 18 months intervention, ACHIEVE participants engaged in three group exercise sessions per week that gradually progressed to reach 40 min of moderate intensity physical activity with 10 min of warm-up and cool-down. In months one through six of the ACHIEVE intervention, group exercise classes were delivered by a study interventionist or certified exercise instructor. In months seven through eighteen, psychiatric rehabilitation program staff delivered one (months seven-12) or two (months 13–18) of the group exercise sessions, with support from a video, developed by study staff, that guided the content and pacing of the exercise class.

Participants were asked to filled out a simple tracking tool to monitor their diet and physical activity behaviors outside of the ACHIEVE intervention. In addition to group and individual sessions, regular weigh-ins with the study interventionist were recommended to support intervention goals. Intervention participation was reinforced with a point system. To receive a point for participation in a weight management group or individual visit, a participant had to attend the entire session. To receive a point for participation in group exercise, a participant had to be standing and engaged in the entire session. Points could be traded for small prizes, e.g., athletic socks or headphones.

## Achieve randomized clinical trial intervention delivery

The ACHIEVE intervention was designed for delivery by an interventionist with a bachelor's degree in a health-related field and by a certified exercise instructor. Staff received significant in-person initial and ongoing training on intervention content and motivational interviewing for weight management sessions. A leaders' guide and manualized procedures were used to ensure standardization of the intervention delivery. Staff were observed regularly to measure intervention fidelity. As noted above, psychiatric rehabilitation program staff delivered video-assisted group exercise sessions in months six through 18 of the study.

## Scaling the achieve intervention

The majority of interventions shown to improve health outcomes in RCTs are never widely implemented. In addition, the effectiveness of interventions often diminishes outside the high-resource, controlled environment of RCTs. This voltage drop in intervention effectiveness is particularly relevant for complex, multi-component interventions—like ACHIEVE—that can be challenging to implement with high-fidelity and the necessary level of intensity in real-world settings.

In a 2017 paper, Gregory Aarons and colleagues presented a framework for scaling up interventions, like ACHIEVE, which have been shown to be effective in RCTs but not yet widely implemented outside of the trial context (29). The authors helpfully distinguish between the concepts of “scale-up,” which they define as efforts to expand an evidence-based intervention to similar settings and populations as those in which the trial was conducted, and “scale-out.” Scale-out is defined as expanding an evidence-based intervention shown to be effective in one setting/population to a different setting and/or population.

In the context of ACHIEVE, scale-up would involve implementation of the intervention in psychiatric rehabilitation programs with similar staffing, programming, and client populations as those in the state of Maryland [while psychiatric rehabilitation programs exist in all 50 U.S. states, they take different forms, e.g., as day programs or “Clubhouses” (30)]. Scale-out of ACHIEVE might involve implementation of ACHIEVE in the outpatient mental health clinic, Assertive Community Treatment (ACT) or residential rehabilitation settings.

In the remainder of this piece, we propose considerations for ACHIEVE scale-up and scale-out. For both, we focus on two overarching steps: (1) adaptation of the ACHIEVE behavioral weight loss intervention and (2) development of implementation strategies to support scale-up/out. The existence of other effective behavioral weight loss interventions for people with SMI including STRIDE (27) and InSHAPE, (26, 31) which share some core principles (e.g., tailoring to address cognitive deficits in SMI) but include varying components, could be an asset to scale-up/out efforts as adaptations to the components of one intervention could be informed by components of other effective interventions. Similarly, some implementation strategies for bringing behavioral weight loss interventions for people with SMI to scale may be intervention or setting-specific, but others are likely relevant across interventions. Strategies shown to result in high-fidelity implementation of the intervention, high rates of consumer participation, and weight loss in the population with SMI should be prioritized for broader use.

## Adaptation of the achieve behavioral weight loss intervention

### Intervention adaptation for scale-up

For scale-up of the ACHIEVE intervention to psychiatric rehabilitation programs similar to those included in the RCT, intervention adaptations should focus on facilitating intervention delivery by psychiatric rehabilitation program staff. Core intervention components (Table 1) should be retained, but changes to intervention contact schedule and adaptations and/or enhancements to delivery format are likely needed in order to enable the psychiatric rehabilitation program staff to deliver the sessions.

For example, the RCT-tested intervention included a total of 150 min per week of group aerobic exercise, delivered in three 50 min sessions, as well as weekly 45 min weight management sessions. For scale-up, the type (group aerobic exercise and weight management) and intensity of these intervention components should remain largely consistent, but the delivery schedule could shift to three 60 min combined weight management/exercise sessions per week, or another schedule that would be more efficient for each psychiatric rehabilitation program.

In addition, adaptations/enhancements to delivery format of the ACHIEVE intervention components are likely needed to support scale-up. For example, while study team interventionists delivered in-person group weight management sessions in the RCT, psychiatric rehabilitation program staff potentially could deliver group sessions supported by videos that are designed to alleviate some of the challenges PRP staff may face when implementing behavioral weight loss programs. These videos could be developed by the study team, and then the psychiatric rehabilitation program staff could utilize a curriculum that blends these tailored videos with the in-person interactive group weight loss sessions.

### Intervention adaptation for scale-out

The scale-up adaptations focused on facilitating intervention delivery by mental health program staff are also relevant for scale-out of the ACHIEVE intervention to different types of mental health programs. However, for scale-out, we suspect that changes to core components of the ACHIEVE intervention may also be required as described below.

For example, outpatient mental health clinic settings, many of which already deliver group and individual counseling, may be well-equipped to deliver to weight-management components of the ACHIEVE intervention. However, they may be less able, due to space and workflow constraints (i.e., shorter office-based clinical visits as opposed to several hour-long psychosocial rehabilitation programs), to deliver the group exercise component of ACHIEVE. The intervention could be adapted in multiple ways to address this issue. Exercise sessions could be shortened and paired with weight loss sessions in order to fit within standard programming time slots at outpatient clinics. Clinics could partner with personal trainers to provide individualized exercise coaching for participants, a strategy shown to be effective in the InSHAPE intervention (26, 31). Additional strategies could target other activities throughout the day, outside of the outpatient clinic setting, such as a pedometer/walking program and exercise videos to use at home.

Although the ACHIEVE intervention was developed and tested in a diverse group of consumers with SMI, changes may also need to be made to improve salience to specific sub-populations with SMI. For example, the intervention has not been translated into other languages. Translation of the ACHIEVE intervention into pediatric patients with SMI may also require modifications.

### Development of implementation strategies

#### Implementation strategies to support scale-up

A multi-component implementation strategy (32) is needed to accomplish widespread scale-up of ACHIEVE to psychiatric rehabilitation programs. Within that multi-component strategy, we propose four key groups of strategies for consideration: staff capacity building strategies, leadership engagement strategies, organizational change strategies, and policy strategies.

Staff capacity building strategies are critical for ACHIEVE scale-up. While the RCT was performed in psychiatric rehabilitation programs, the ACHIEVE intervention was largely, with the exception of some group exercise classes, delivered by trained study team interventionists. To effectively implement ACHIEVE, program staff need training not only in delivery of the ACHIEVE intervention but also in motivational interviewing, the counseling technique used in ACHIEVE. Staff trainings on these topics will need to be developed and disseminated in a manner that allows busy staff members to participate. This training is a challenge given that typical motivational interviewing training involves, at a minimum, 2 days in-person workshops and repeated post-workshop feedback and coaching. Virtual training modalities and incentives for staff to participate, such as continuing education credits, need to be developed. In addition, performance coaching and audit-and-feedback strategies could support staff members' ability to deliver the ACHIEVE intervention. To ensure that the staff members delivering ACHIEVE are capable of doing so with high fidelity, a certification program could be created where staff become certified ACHIEVE interventionists after completing a specified level of training and demonstrating competency in intervention delivery, similar to what is available with Center for Disease Control's National Diabetes Prevention Program (DPP) (33–35).

Leadership engagement strategies are needed to facilitate leaders' adopting and providing ongoing support for ACHIEVE implementation at their organization. An example of a potential strategy within this domain is the use of an opinion leader(s) (32)–i.e., a prominent psychiatric program rehabilitation leader at the local, state or national l level—to promote ACHIEVE.

Organizational strategies are needed to help psychiatric rehabilitation programs foster a culture that is supportive of ACHIEVE implementation and of good physical health more broadly. For example, many psychiatric rehabilitation programs serve food and could benefit from guidance on how to improve the healthfulness of their options. In addition, programs could benefit from guidance on creating safe places for group exercise. Organizational policies, e.g., tobacco-free policies and policies prohibiting vending machines offering sugar drinks, are also an important strategy in this domain.

Policy strategies are a key component of scale-up. Critically, a financing mechanism(s) is needed to allow psychiatric rehabilitation programs to cover the cost of ACHIEVE implementation. In Maryland, both weight management sessions and group exercise are reimbursable by Medicaid as psychiatric rehabilitation services; if this financing mechanism does not exist in other state Medicaid programs, it would need to be created. Creation of a parallel reimbursement mechanism in Medicare and among private insurers would further facilitate adoption and implementation. Medicare currently covers the Diabetes Prevention Program (33–36), setting a precedent for coverage of weight loss interventions, and the Medicare obesity counseling benefit (37) could potentially be expanded to cover the weight management components of the ACHIEVE intervention.

Financing mechanisms for implementation strategies also need to be developed, e.g., if research shows that external facilitation and audit-and-feedback are needed to support high-fidelity implementation of ACHIEVE by program staff, mechanisms to pay for these strategies need to be created. Other types of policies can also support ACHIEVE scale-up, for example accreditation standards requiring that programs have a certified ACHIEVE trainer on staff. Performance metrics are increasingly tied to payment through healthcare financing and delivery system reforms such Accountable Care Organizations (ACOs). Tying a metric measuring the proportion of overweight and obese people with SMI who lose weight through an evidence-based behavioral weight loss intervention like ACHIEVE to intervention reimbursement by insurers could facilitate implementation.

#### Implementation strategies to support scale-out

In some cases, the strategies developed for scale-up could also be used, with no or minimal adaptation, for scale-out. For example, an online motivational interviewing and weight management training program developed for psychiatric rehabilitation program staff could also be used for staff at other types of mental health programs. In other cases, new implementation strategies may need to be developed for scale-out, e.g., financing mechanisms may differ depending upon an organization's funding sources.

For scale-out, ACHIEVE intervention components may need to be implemented across different organizations, e.g., the outpatient clinic/community fitness center example above. Thus, implementation strategies to support the development of partnerships are likely needed for successful scale-out, e.g., development of model memorandum of understanding and financing strategies that organizations can use to facilitate effective collaborations.

### Future research to support scale-up/out

The key research questions around both scale-up and scale-out of ACHIEVE are which intervention adaptations and implementation strategies result in (1) adoption and sustained, high-fidelity implementation of the intervention in mental health programs and (2) clinically significantly weight loss among participants.

Aarons et al. describe a spectrum of potential research designs for scale-up/out (29). On one end of the spectrum are designs for scale-up scenarios where the setting and population are very similar to those in the RCT, and minimal adaptations to the RCT-tested intervention have been made. For these types of scenarios, a research design, like a hybrid type III trial design that focuses primary on evaluating implementation strategies, may be appropriate. One the other end of the spectrum is a scale-out scenario in which new implementation strategies have been developed and significant adaptations to the intervention have been made. In this scenario, a research design like a hybrid type II trial design that simultaneously tests both implementation strategy and intervention effectiveness (38) may be needed.

We posit that research to test implementation strategies for ACHIEVE scale-up and scale-out, which have not yet been developed or evaluated, should be prioritized. Given the barriers to high-fidelity implementation of complex interventions like ACHIEVE in often under-resourced mental health programs, implementation strategies should be shown to be effective through rigorous randomized trials before resources are invested in broad scale-up/out efforts. Financing mechanisms to support ACHIEVE scale-up and scale-out need to be developed and evaluated, and the cost-effectiveness of scaling ACHIEVE should also be studied. For both scale-up and scale-out, the degree to which the adapted ACHIEVE intervention differs from the original will be important in terms of deciding whether evaluation of weight change among participants with SMI is needed.

## Author contributions

All authors contributed to the ideas presented in this Perspective and reviewed the final version of the article EM led manuscript writing.

### Conflict of interest statement

The authors declare that the research was conducted in the absence of any commercial or financial relationships that could be construed as a potential conflict of interest.

## References

[1] Olfson M Gerhard T Huang C Crystal S Stroup TS. Premature mortality among adults with schizophrenia in the United States. JAMA Psychiatry (2015) 72:1172–81. 10.1001/jamapsychiatry.2015.1737 26509694

[2] Roshanaei-Moghaddam BMD Katon WMD. Premature mortality from general medical illnesses among persons with bipolar disorder: a review. Psychiatr Serv. (2009) 60:147–56. 10.1176/ps.2009.60.2.147 19176408

[3] Osborn DP Levy G Nazareth I Petersen I Islam A King MB. Relative risk of cardiovascular and cancer mortality in people with severe mental illness from the United Kingdom's General Practice Rsearch Database. Arch Gen Psychiatry (2007) 64:242–9. 10.1001/archpsyc.64.2.242 17283292

[4] Ösby U Brandt L Correia N Ekbom A Sparén P. Excess mortality in bipolar and unipolar disorder in Sweden. Arch Gen Psychiatry (2001) 58:844–50. 10.1001/archpsyc.58.9.844 11545667

[5] Ösby U Correia N Brandt L Ekbom A Sparén P. Mortality and causes of death in schizophrenia in Stockholm county, Sweden. Schizophr Res. (2000) 45:21–8. 10.1016/S0920-9964(99)00191-7 10978869

[6] Saha S Chant D McGrath J. A systematic review of mortality in schizophrenia: is the differential mortality gap worsening over time? Arch Gen Psychiatry (2007) 64:1123–31. 10.1001/archpsyc.64.10.1123 17909124

[7] Janssen EM McGinty EE Azrin ST Juliano-Bult D Daumit GL. Review of the evidence: prevalence of medical conditions in the United States population with serious mental illness. Gen Hosp Psychiatry (2015) 37:199–222. 10.1016/j.genhosppsych.2015.03.004 25881768 PMC4663043

[8] Casagrande SS Anderson CA Dalcin A Appel LJ Jerome GJ Dickerson FB. Dietary intake of adults with serious mental illness. Psychiatr Rehabil J. (2011) 35:137–40. 10.2975/35.2.2011.137.140 22020844 PMC7199388

[9] Gupta A Craig TKJ. Diet, smoking and cardiovascular risk in schizophrenia in high and low care supported housing. Epidemiol Psychiatr Sci. (2009) 18:200–7. 10.1017/S1121189X00000476 20034197

[10] Henderson DC Borba CP Daley TB Boxill R Nguyen DD Culhane MA. Dietary intake profile of patients with schizophrenia. Annals of clinical psychiatry (2006) 18:99–105. 10.1080/10401230600614538 16754415

[11] McCreadie R Macdonald E Blacklock C Tilak-Singh D Wiles D Halliday J. Dietary intake of schizophrenic patients in Nithsdale, Scotland: case-control study. BMJ (1998) 317:784–5. 10.1136/bmj.317.7161.784 9740565 PMC28668

[12] Osborn DPJ Nazareth I King MB. Physical activity, dietary habits and Coronary Heart Disease risk factor knowledge amongst people with severe mental illness. Soc Psychiatry Psychiatr Epidemiol. (2007) 42:787–93. 10.1007/s00127-007-0247-3 17721669

[13] Vancampfort D Knapen J Probst M Scheewe T Remans S De Hert M. A systematic review of correlates of physical activity in patients with schizophrenia. Acta Psychiatr Scand. (2012) 125:352–62. 10.1111/j.1600-0447.2011.01814.x 22176559

[14] Vancampfort D Probst M Scheewe T Maurissen K Sweers K Knapen J. Lack of physical activity during leisure time contributes to an impaired health related quality of life in patients with schizophrenia. Schizophr Res. (2011) 129:122–7. 10.1016/j.schres.2011.03.018 21493044

[15] Wolff E Gaudlitz K von Lindenberger BL Plag J Heinz A Strohle A. Exercise and physical activity in mental disorders. Eur Arch Psychiatry Clin Neurosci. (2011) 261 (Suppl. 2):S186–91. 10.1007/s00406-011-0254-y 21935629

[16] Daumit G Goldberg R Anthony C Dickerson F Brown CH Kreyenbuhl J. Physical activity patterns in adults with severe mental illness. J Nerv Mental Dis. (2005) 193:641–6. 10.1097/01.nmd.0000180737.85895.60 16208158

[17] McGinty EE Daumit GL Epidemiology of obesity. Psychiatr Ann. (2011) 41:484–8. 10.1016/j.gtc.2009.12.014

[18] McEvoy JP Meyer JM Goff DC Nasrallah HA Davis SM Sullivan L. Prevalence of the metabolic syndrome in patients with schizophrenia: baseline results from the Clinical Antipsychotic Trials of Intervention Effectiveness (CATIE) Schizophrenia Trial and comparison with national estimates from NHANES III. Schizophr Res. (2005) 80:19–32. 10.1016/j.schres.2005.07.014 16137860

[19] McIntyre RS Jerrell JM. Metabolic and cardiovascular adverse events associated with antipsychotic treatment in children and adolescents. Arch PediatrAdoles Med. (2008) 162:929–35. 10.1001/archpedi.162.10.929 18838645

[20] Melkersson K Dahl ML. Adverse metabolic effects associated with atypical antipsychotics: literature review and clinical implications. Drugs (2004) 64:701–23. 10.2165/00003495-200464070-00003 15025545

[21] Morrato EH Cuffel B Newcomer JW Lombardo I Kamat S Barron J. Metabolic risk status and second-generation antipsychotic drug selection: a retrospective study of commercially insured patients. J Clin Psychopharmacol. (2009) 29:26–32. 10.1097/JCP.0b013e31819294cb 19142103

[22] McGinty EE Baller J Azrin ST Juliano-Bult D Daumit GL. Interventions to address medical conditions and health-risk behaviors among persons with serious mental illness: a comprehensive review. Schizophr Bull. (2015) 42:96–124. 10.1016/j.schres.2015.04.010 26221050 PMC4681556

[23] Daumit GL Dickerson FB Wang NY Dalcin A Jerome GJ Anderson CAM. A behavioral weight loss intervention in persons with serious mental illness. NEJM (2013) 368:1594–602. 10.1056/NEJMoa1214530 23517118 PMC3743095

[24] Casagrande SS Jerome GJ Dalcin AT Dickerson FB Anderson CA Appel LJ. Randomized trial of achieving healthy lifestyles in psychiatric rehabilitation: the ACHIEVE trial. BMC Psychiatry (2010) 10:108. 10.1186/1471-244X-10-108 21144025 PMC3016313

[25] Siddiqui M Cooper LA Appel LJ Yu A Charleston J Gennusa J. Recruitment and enrollment of African Americans and Caucasians in a health promotion trial for persons with serious mental illness. Ethnic Dis. (2015) 25:72. 25812255 PMC4663046

[26] Bartels SJ Pratt SI Aschbrenner KA Barre LK Jue K Wolfe RS. Clinically significant improved fitness and weight loss among overweight persons with serious mental illness. Psychiatr Serv. (2013) 64:729–36. 10.1176/appi.ps.003622012 23677386 PMC5662189

[27] Green CA Yarborough BJH Leo MC Yarborough MT Stumbo SP Janoff SL. The STRIDE weight loss and lifestyle intervention for individuals taking antipsychotic medications: a randomized trial. Am J Psychiatry (2014) 172:71–81. 10.1176/appi.ajp.2014.14020173 25219423 PMC4282602

[28] Appel L Champagne C Harsha D Cooper LS Obarzanek E Elmer PJ. Effects of comprehensive lifestyle modification on blood pressure control: main results of the PREMIER clinical trial. writing group of the premier collaborative research group. JAMA (2003) 289:2083–2093. 10.1001/jama.289.16.2083 12709466

[29] Aarons GA Sklar M Mustanski B Benbow N Brown CH “Scaling-out” evidence-based interventions to new populations or new health care delivery systems. Implement Sci. (2017) 12:111 10.1186/s13012-017-0640-6 28877746 PMC5588712

[30] Psychiatric Rehabilitation Association Individual Directory (2017). Available online at: https://netforum.avectra.com/eweb/DynamicPage.aspx?Site=praandprf&WebCode=IndSearch (Accessed December 13, 2017).

[31] Bartels SJ Pratt SI Aschbrenner KA Barre LK Naslund JA Wolfe R. Pragmatic replication trial of health promotion coaching for obesity in serious mental illness and maintenance of outcomes. Am J Psychiatry (2015) 172:344–52. 10.1176/appi.ajp.2014.14030357 25827032 PMC4537796

[32] Powell BJ Waltz TJ Chinman MJ Damschroder LJ Smith JL Matthieu MM. A refined compilation of implementation strategies: results from the Expert Recommendations for Implementing Change (ERIC) project. Implement Sci. (2015) 10:21. 10.1186/s13012-015-0209-1 25889199 PMC4328074

[33] The Diabetes Prevention Program (DPP) Research Group The Diabetes Prevention Program (DPP): description of lifestyle intervention. Diabetes Care (2002) 25:2165–71. 10.2337/diacare.25.12.2165 12453955 PMC1282458

[34] Group DPPR 10-year follow-up of diabetes incidence and weight loss in the diabetes prevention program outcomes study. Lancet (2009) 374:1677–86. 10.1016/S0140-6736(09)61457-4 19878986 PMC3135022

[35] Centers for Disease Control and Prevention National Diabetes Prevention Program. (2018). Available online at: https://www.cdc.gov/diabetes/prevention/index.html (Accessed August 31, 2018).

[36] Centers for Medicare and Medicaid Services Medicare Diabetes Prevention Program (MDPP) Expanded Model (2018). Available online at: https://innovation.cms.gov/initiatives/medicare-diabetes-prevention-program/ (Accessed August 31, 2018).

[37] Batsis JA Bynum JPW. Uptake of the centers for medicare and medicaid obesity benefit: 2012–2013. Obesity (2016) 24:1983–8. 10.1002/oby.21578 27465909 PMC5003721

[38] Curran GM Bauer M Mittman B Pyne JM Stetler C. Effectiveness-implementation hybrid designs: combining elements of clinical effectiveness and implementation research to enhance public health impact. Medical Care (2012) 50:217–26. 10.1097/MLR.0b013e3182408812 22310560 PMC3731143

